# Integrating untargeted metabolomics and deep learning approaches to identify specific metabolic signatures and new mechanisms in unstable plaques

**DOI:** 10.3389/fcvm.2026.1646067

**Published:** 2026-05-12

**Authors:** Jia-Qi Ma, Lu Wang, Xiao-Peng Qu, Yue Zhang, Li-Jia Song, Guo-Dong Gao, Chao Wang, Long-Long Zheng, Qi-Xing Fang, Yan Qu, Liang-Liang Shen, Bei Liu

**Affiliations:** 1 Department of Neurosurgery, Tangdu Hospital, Airforce Military Medical University, Xi'an, Shaanxi, China; 2 College of Life Sciences, Northwest University, Xi'an, Shaanxi, China; 3 Department of Pediatrics, Tangdu Hospital, Airforce Military Medical University, Xi'an, China; 4 Department of Biochemistry and Molecular Biology, Basic Medical Science Academy, Airforce Military Medical University, Xi'an, Shaanxi, China

**Keywords:** biomarkers, carotid artery, machine learning, metabolomics, unstable plaque

## Abstract

**Background:**

Unstable carotid artery plaques are an important risk factor for ischemic stroke, and their clinical prognosis is poor. The present study to systematically investigate the metabolic changes of carotid plaques and use machine learning methods to identify and screen metabolic biomarkers in unstable carotid plaques for helping diagnosis of stroke risk caused by unstable plaques.

**Method:**

A non-targeted metabolomics analysis was performed on 67 cases (40 stable and 27 unstable) of carotid artery plaques. Specific metabolic signatures were identified in unstable plaques. Four machine learning algorithms, including random forest (RF), support vector machine (SVM), least absolute shrinkage and selection operator (LASSO), and logistic regression (LR), were used to construct feature analysis models for unstable carotid artery plaques and predict the associated metabolic biomarkers.

**Results:**

A total of 98 metabolites significantly differentially associated with unstable plaques were identified. Kyoto Encyclopedia of Genes and Genomes (KEGG) enrichment analysis revealed that the cGMP-PKG signaling pathway, glucagon signaling pathway, central carbon metabolism in cancer, and lipolysis regulation in adipocytes are metabolic pathways significantly associated with unstable plaques. The network diagram of metabolites and metabolic pathways revealed the relationship between 43 metabolites and their corresponding pathways. Furthermore, some metabolites that may serve as biomarkers for unstable plaques were screened.

**Conclusion:**

Different metabolite patterns associated with unstable plaque tissue were identified and characterized. This study identified some potential metabolic biomarkers significantly associated with unstable carotid artery plaques, which can predict metabolic products and further improve the prediction of stroke risk in unstable plaques.

## 1 Introduction

Atherosclerotic plaques can cause carotid artery lumen stenosis, reduce blood flow to brain cells, or rupture and become dislodged, leading to stroke events. Stroke is one of the causes of adult disability (1). Early lesion symptoms are not obvious and rarely receive attention in the process of carotid artery plaque formation (2). Intervention, such as carotid endarterectomy, typically takes place when the degree of carotid artery stenosis is high (3). However, cerebrovascular events may also occur in patients with low-grade carotid artery stenosis (<30%) and no other obvious causes of stroke (4). Therefore, early detection of carotid atherosclerotic disease and reliable identification of plaque instability are of great significance for stroke prevention.

Carotid artery plaques are divided into stable and unstable plaques (5). Unstable carotid artery plaques are characterized by thin fibrous caps and large lipid cores (1), which are the initial changes leading to plaque rupture (5, 6). Advances in imaging technology have allowed us to detect carotid plaque features, and Doppler ultrasound imaging can identify stable and unstable plaques (7, 8). Unstable plaques include hypoechoic lipid plaques, mixed plaques with significantly uneven echoes, ulcerative plaques with uneven surfaces, and bleeding plaques without internal echoes (9). In addition, there are reports suggesting that metabolic disorders of carbohydrates, lipids, and amino acids can lead to plaque instability. Therefore, the structure, composition, and production of metabolites within plaques may be more direct markers of plaque rupture compared to the degree of luminal stenosis and stroke risk. Thus, the biological metabolic basis of unstable plaques needs to be explored further (10).

Metabolomics is an emerging omics technique that involves detailed quantification of small molecule metabolic markers in biological samples that can then be used to identify new biomarkers (11), diagnose and monitor diseases, and characterize metabolic pathways in disease pathogenesis (12). At present, the analysis of related metabolites in human atherosclerotic plaque tissue using metabolomics has been reported (10), but scarce studies have focused on the metabolomic characterization of stable/unstable plaques. Our research incorporates clinical data and applies machine learning techniques.

To comprehensively study the metabolic characteristics of unstable carotid plaques, the present study used multivariate analysis to identify 98 potential metabolic biomarkers of unstable plaques based on untargeted metabolomics. Metabolic pathway enrichment analysis was performed to explore the metabolic pathways and metabolites enriched in unstable plaques. Subsequently, four machine learning algorithms, including RF, SVM, LASSO, and LR, were used to further identify metabolic biomarkers associated with unstable plaques, which can further improve the prediction of stroke risk in unstable plaques.

## 2 Methods

### 2.1. Study design and participants

The present case-control study was carried out at the Xi'an Tangdu Hospital as a part of two concurrent multicenter cohort studies (registered on ClinicalTrails.gov, NCT06027463 and NCT06120478). A total of 67 patients with carotid artery stenosis were included in the study between June 2022 and August 2023. All participants met the following selection criteria: (1) patients over 18 years old diagnosed with carotid artery stenosis and receiving carotid endarterectomy (CEA) for the first time; (2) preoperative imaging examination confirmed carotid artery stenosis of >70%; (3) informed consent form was provided; and (4) follow-up after surgery was performed at the treatment institution. Participants with the following characteristics were excluded from the study: (1) age of <18 or >85 years; (2) refractory hypertension; (3) severe bleeding tendency; (4) complete occlusion of the responsible carotid artery (100%); (5) multiple stenosis of the unilateral carotid artery beyond the surgical range; (6) untreated intracranial large aneurysm; (7) severe surgical contraindications; and (8) other malignant diseases, such as malignant tumors, or expected death within one year. The study was approved by the Medical Ethics Committee of Tangdu Hospital (K202211-29). All carotid artery plaques were divided into stable and unstable plaque groups after combining the patients' carotid artery ultrasound examination results. The unstable group included ulcerative, hemorrhagic, and echolucent plaques, while the stable group included echogenic plaques. Mixed echogenic plaques and plaques that were difficult to classify were excluded. The plaque characteristics were reviewed and confirmed by two senior professional carotid and cerebrovascular ultrasound physicians. A total of 67 patients were included in the final metabolomics analysis: 40 in the stable group and 27 in the unstable group. The patients and/or their caregivers signed informed consent in accordance with the standards of the Declaration of Helsinki.

### 2.2. Sample collection and preparation

Carotid artery plaque tissue was used to identify metabolites in the present study. Carotid artery plaques obtained during carotid endarterectomy were immediately processed and stored in polypropylene tubes at −80 ℃. Then, 50 mg of solid sample was added to a 2-mL centrifuge tube together with a grinding bead 6 mm in diameter. Next, 400 μL of extraction solution [methanol: water = 4:1 (v:v)] containing 0.02 mg/mL of the internal standard (L-2-chlorophenylalanine) was used for metabolite extraction. Samples were ground using the Wonbio-96c (Shanghai wanbo biotechnology Co., Ltd.) frozen tissue grinder for 6 min (−10 °C, 50 Hz), followed by low-temperature ultrasonic extraction for 30 min (5 °C, 40 kHz). The samples were then kept at −20 °C for 30 min, after which they were centrifuged for 15 min (4 °C, 13,000 g). The resulting supernatant was transferred to the injection vial for liquid chromatography-tandem mass spectrometry (LC-MS/MS) analysis.

### 2.3 Quality control sample

As a part of the system conditioning and quality control (QC) process, a pooled QC sample was prepared by mixing equal volumes of all samples. The QC samples were disposed and tested in the same manner as the analytic samples. The whole sample set was injected at regular intervals (every 5–15 samples) in order to monitor analysis stability.

### 2.4 Ultra-high-performance liquid chromatography tandem mass spectrometry (UHPLC-MS/MS) analysis

The LC-MS/MS analysis was conducted using a Thermo UHPLC-Q Exactive HF-X system equipped with an ACQUITY HSS T3 column (100 mm × 2.1 mm i.d., 1.8 μm; Waters, USA) at Majorbio Bio-Pharm Technology Co. Ltd. (Shanghai, China). The mobile phases consisted of 0.1% formic acid in water:acetonitrile (95:5, v/v; solvent A) and 0.1% formic acid in acetonitrile:isopropanol:water (47.5:47.5, v/v; solvent B). The flow rate was 0.40 mL/min and the column temperature was 40 ℃. The injection volume was 3 μL.

#### 2.4.1 MS conditions

Mass spectrometry data were collected using a Thermo UHPLC-Q Exactive HF-X mass spectrometer equipped with an electrospray ionization (ESI) source operating in positive and negative modes. The optimal conditions were set as follows: source temperature at 425 ℃; sheath gas flow rate at 50 arb; aux gas flow rate at 13 arb; ion-spray voltage floating (ISVF) at −3,500 V in negative mode and 3,500 V in positive mode; and normalized collision energy of 20–40–60 eV rolling for MS/MS. Full MS resolution was 60,000, and MS/MS resolution was 7,500. Data acquisition was performed in the Data-Dependent Acquisition (DDA) mode. The detection was carried out over a mass range of 70–1,050 m/z.

### 2.5 Machine learning models

#### 2.5.1 RF

An RF model was constructed using the Majorbio Cloud Platform (cloud.majorbio.com) to identify biomarkers most likely associated with unstable carotid artery plaques. Receiver operating characteristic (ROC) analysis for the RF models was performed on the same platform.

#### 2.5.2 SVM

ROC curve analysis was used to evaluate the diagnostic performance of potential biomarkers. The SVM classification model and ROC analyses for multiple biomarkers were performed using the Majorbio Cloud Platform (cloud.majorbio.com).

#### 2.5.3 LASSO and logistic regression

The LASSO regression is a penalty function that finds the most representative molecules by differentially scaling down the variables. LASSO regression in the present study was used to screen metabolites specifically expressed in unstable carotid artery plaques. Logistic stepwise regression was utilized to construct a diagnostic model. In addition, ROC curves were used to assess the model's ability to differentiate between stable and unstable carotid artery plaque groups.

### 2.6 Data analysis and statistics

**Figure 1:**
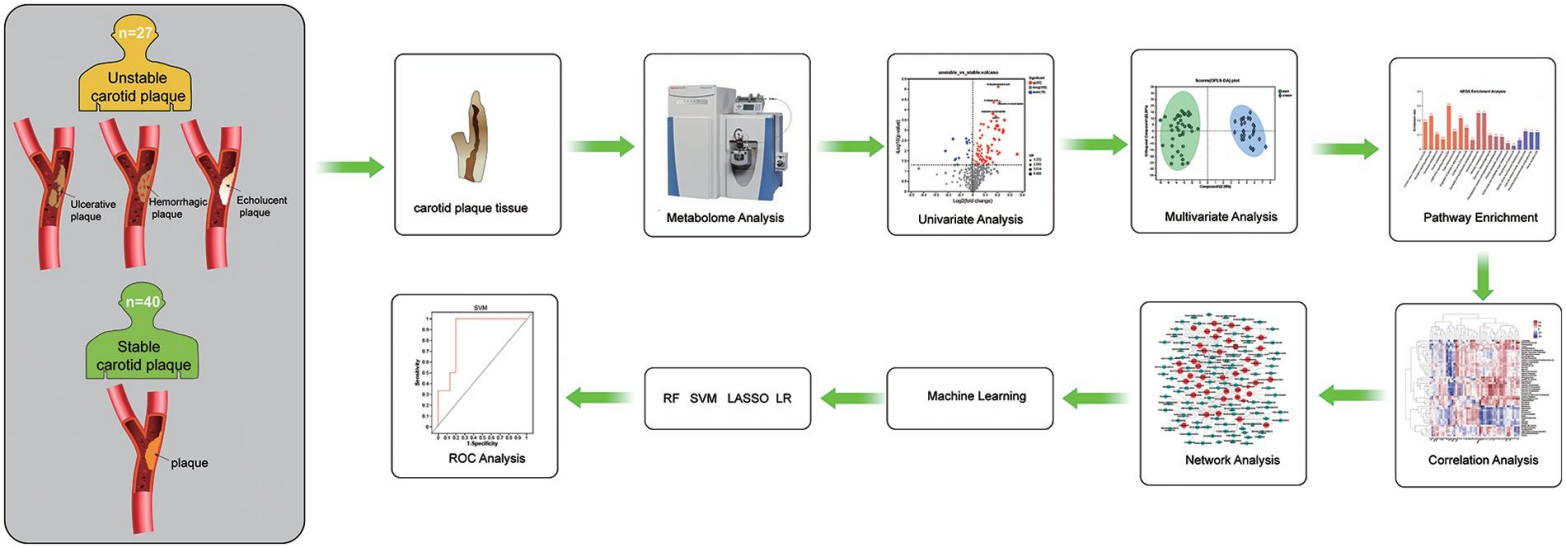
Study design and data analysis workflow. RF, Random forest; SVM, Support vector machine.

The study design and data analysis workflow are shown in Figure 1. The LC/MS raw data pretreatment was performed using Progenesis QI software (Waters Corporation, Milford, USA). A three-dimensional data matrix in CSV format was then exported. It contained sample information, metabolite name, and mass spectral response intensity. Internal standard peaks, as well as any known false positive peaks (including noise, column bleed, and derivatized reagent peaks), were removed from the data matrix, deredundant, and peak pooled. At the same time, the metabolites were identified by searching the HMDB (http://www.hmdb.ca/), Metlin (https://metlin.scripps.edu/), and Majorbio databases.

The data matrix obtained by searching the databases was uploaded to the Majorbio cloud platform (https://cloud.majorbio.com) for data analysis. The data matrix was then pre-processed. First, at least 80% of the metabolic features detected in any set of samples were retained. After filtering, the minimum value in the data matrix was selected to fill the missing value and each metabolic signature was normalized to the sum. To reduce the errors caused by sample preparation and instrument instability, the response intensities of the sample mass spectrometry peaks were normalized using the sum normalization method to obtain the normalized data matrix. The QC sample variables with a relative standard deviation (RSD) of >30% were excluded and log10 logarithmicized to obtain the final data matrix for subsequent analysis.

Then, the R package “ropls” (version 1.6.2) was used to perform principal component analysis (PCA), orthogonal least partial squares discriminant analysis (OPLS-DA), and seven-cycle interactive validation evaluating model stability. The metabolites with variable importance on projection (VIP) > 1 and *p* < 0.05 were determined to be significantly different based on the variable importance in the projection (VIP) obtained using the OPLS-DA model and the *p*-value generated by Student's *t* test.

Differential metabolites among two groups were mapped into their biochemical pathways using metabolic enrichment and pathway analysis based on the KEGG database (http://www.genome.jp/kegg/). Cytoscape version 3.7.0 was used to create a network diagram of metabolites and their related pathways. These metabolites were classified according to the pathways they were involved in or the functions they performed. Enrichment analysis was used to evaluate a group of metabolites in a function node. The annotation analysis of a single metabolite was developed into annotation analysis of a group of metabolites. Python package “scipy.stats” (https://docs.scipy.org/doc/scipy/) was used to perform enrichment analysis to obtain the most relevant biological pathways for experimental treatments.

Statistical analyses were performed using SPSS 25.0 (IBM Corporation, New York, NY, USA). Two-tailed *p* < 0.05 was considered statistically significant.

## 3 Results

### 3.1 Patients' clinical characteristics and subsequent analysis

In order to explore the differences in metabolic characteristics of stable and unstable plaques in patients with carotid artery stenosis, a total of 67 subjects were selected and divided into stable (40 cases) and unstable (27 cases) plaque groups according to preoperative carotid artery ultrasound results. A total of 49 general clinical characteristics of the subjects are shown in Supplementary Table S1. There are certain differences in biological gender, TIA, hypertension, and levels of GLO and TP between the two groups (*P* < 0.05). Next, metabolomics analysis was performed on plaque tissue samples obtained during carotid endarterectomy. A total of 98 differential metabolites were identified, including 82 up-regulated and 16 down-regulated metabolites. Finally, machine learning algorithms were used to analyze the differential expression of metabolites in two groups of plaque tissues, select potential metabolic biomarkers, and provide more meaningful targets for prediction of unstable plaques (Figure 1).

### 3.2 Metabolic biomarker identification

**Figure 2:**
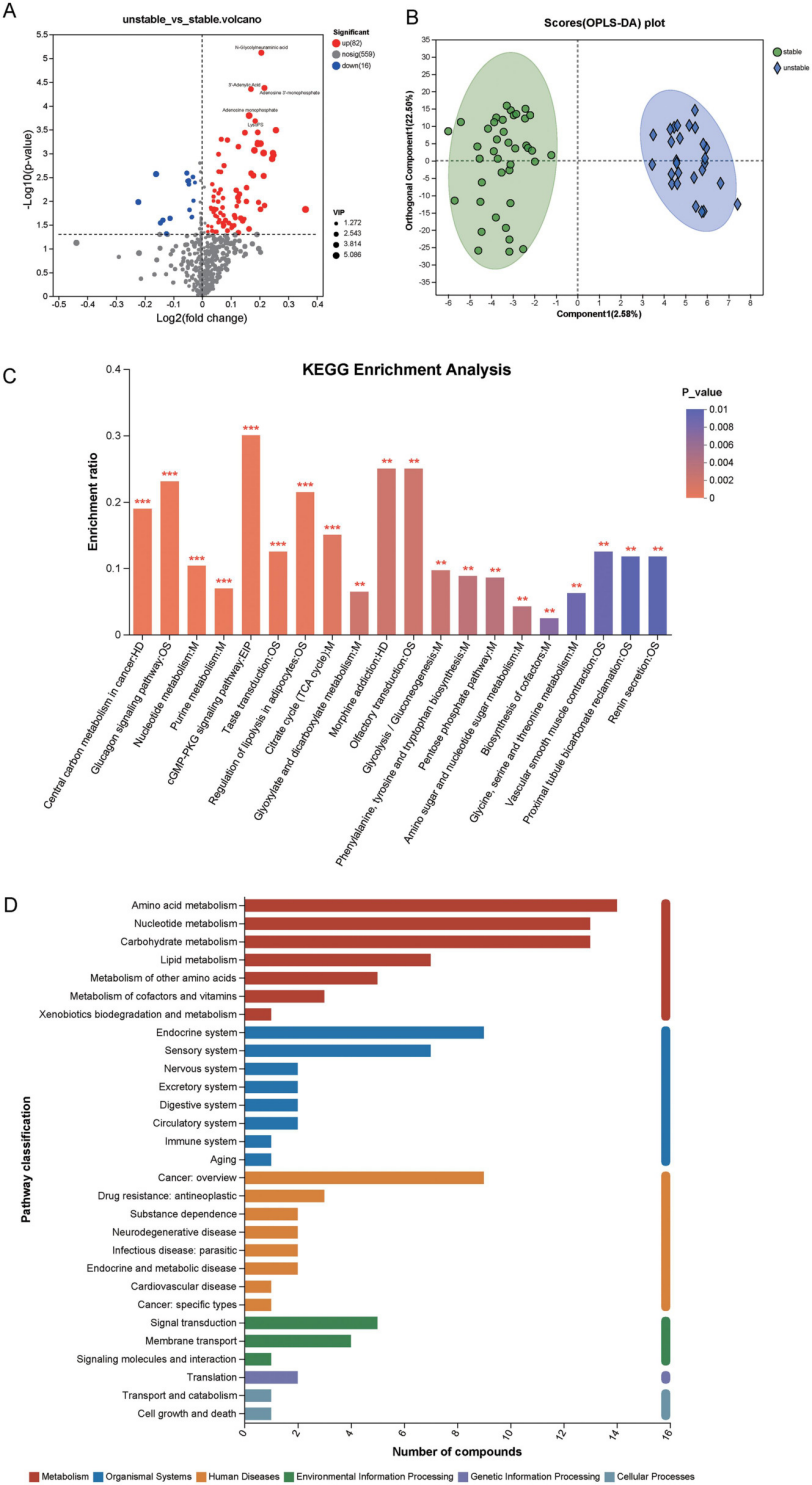
Identification of metabolic biomarkers and related pathways associated with unstable carotid artery plaques. **(A)** Volcano plots showing the differential metabolite screening by fold change (>1) and *p*-value (<0.05) using *t*-tests. **(B)** Score plot of OPLS-DA in the unstable and stable groups; **(C)** Metabolic pathway enrichment study of differentially presented between the unstable group and stable group. The abscissa represents the pathway name. The ordinate represents the enrichment rate, which represents the ratio of the number of metabolites enriched in the pathway to the number of metabolites annotated in the pathway. The color gradient of the column represents the significance of enrichment. The darker the color, the more significant the enrichment of the KEGG term. Using Fisher's test to calculate *p*-value. *P* value or FDR < 0.001 is marked with ***, *P* value or FDR < 0.01 is marked with **, and *P* value or FDR < 0.05 is marked with *; **(D)** Annotated diagram of KEGG functional pathway enrichment. The vertical axis represents the secondary classification of KEGG metabolic pathways, and the horizontal axis represents the number of metabolites annotated under this pathway. *n*_unstable plaque_ = 27, *n*_stable plaque_ = 40.

The metabolomics results for carotid plaque tissue samples from 67 patients with carotid artery stenosis were analyzed using bioinformatics tools. As a result, 657 differential metabolites associated with unstable plaques were annotated, of which 387 were up-regulated and 270 were down-regulated in the unstable plaque group (Supplementary Figure 1A). The VIP values were calculated for each metabolite using PCA and OPLS-DA models. Metabolites with VI*P* > 1 were considered potential candidate metabolites. Out of 657 differential metabolites, 98 metabolites with VIP > 1 and *p* < 0.05 were screened, of which 82 were up-regulated and 16 were down-regulated (Figure 2A, Supplementary Table S2).

PCA analysis revealed that the inter group repeatability effect and inter group dispersion were unsatisfactory (Supplementary Figure 1B). Therefore, further calculation of the cumulative R2Y and Q2 values in the OPLS-DA model was used to estimate the goodness of fit and predictive ability of the model. The cumulative R2Y of the OPLS-DA model was 0.950, and the cumulative Q2 was 0.481. The OPLS-DA score chart shows that the stable plaque group is separated from the unstable plaque group (Figure 2B).

### 3.3 Metabolic pathway enrichment analysis

**Figure 3:**
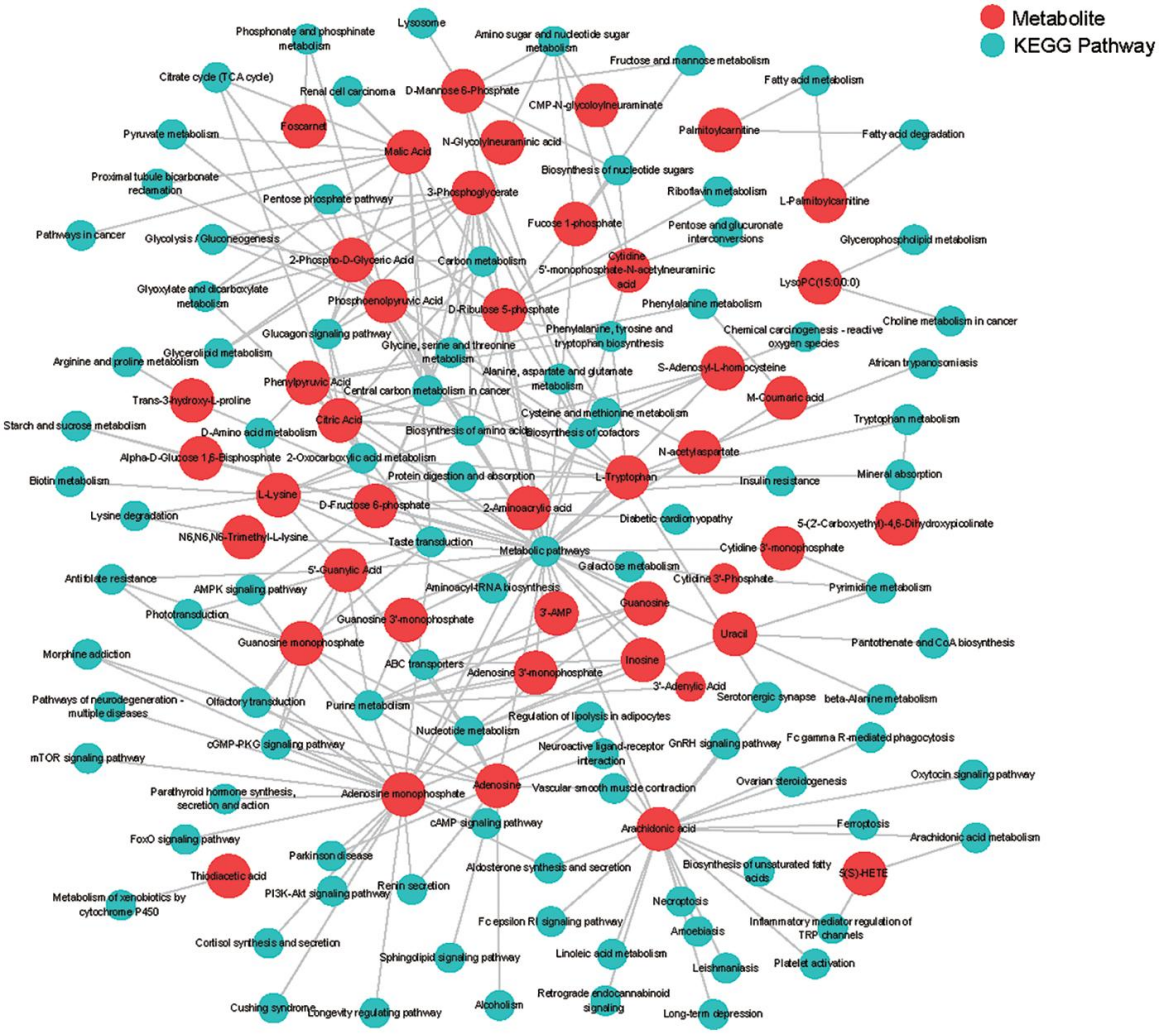
Network analysis of differential metabolites and related metabolic pathways between the unstable plaque group and the stable plaque group.

Metabolic pathway analysis was conducted based on 98 differential metabolites to reveal metabolic pathways associated with unstable plaques. These metabolites were involved in 89 metabolic pathways (Supplementary Table S3). Among the 89 metabolic pathways, 38 had *p* < 0.05. The top 20 of these pathways are shown in Figure 2C. In order to further understand the functions of KEGG enriched pathways, KEGG functional pathway enrichment analysis was conducted on 98 differential metabolites. Six of seven KEGG metabolic pathway categories were enriched, including metabolism, genetic information processing, environmental information processing, cellular processes, organizational systems, and human diseases. Among them, the lipid metabolism pathway was enriched with seven related metabolites (palmitoylcarnitine, LysoPC (15:0/0:0), 2-phospho-D-glyceric acid, L-palmitoylcarnitine, arachidonic acid, 5 (S)-HETE, and 3-phosphoglycerate; Figure 2D, Supplementary Table S4). The search for 98 differential metabolites revealed that only 43 metabolites were present in the KEGG pathway database (Supplementary Table S5). Therefore, the relationship between these 43 metabolites and the pathways they enriched was analyzed and the network of metabolites and metabolic pathways was plotted (Figure 3).

### 3.4 Association between differential metabolites and clinical indicators

**Figure 4:**
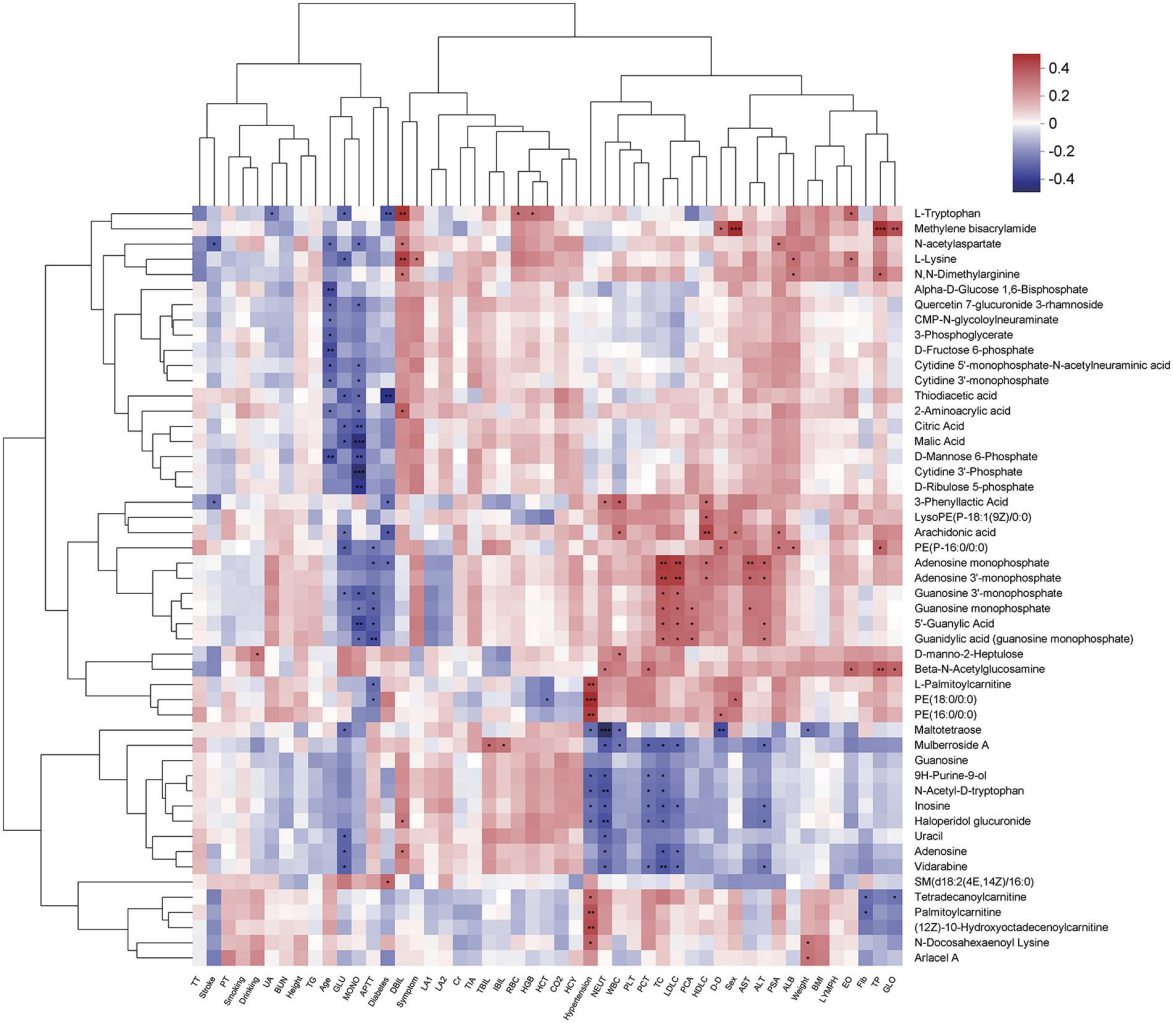
Correlations between top 50 differential metabolites and clinical characteristics of patients. Each row represents the metabolite name, and each column represents the patient's clinical characteristics. Each cell represents the Pearson correlation coefficient between clinical features and metabolites. Red indicates a positive correlation, while purple indicates a negative correlation. *There was a significant correlation between clinical features and metabolites (*P* < 0.05). Using Pearson algorithm to calculate correlation coefficients.

The Pearson correlation coefficient was calculated to investigate the potential relationship between the top 50 differential metabolites and 49 clinical indicators (Supplementary Table S6). The correlation coefficient matrix is shown in Figure 4. Among the 50 metabolites, 15 differential metabolites were associated with MONO, 13 with TC, 12 with GLU and hypertension, 11 with NEUT, 10 with age and LDLC, eight with APTT and ALT, seven with DBIL and PCT, six with diabetes, five with WBC and HDLC, four with D-D and TP, three with PCA, sex, AST, PSA, ALB, weight, EO, and GLO, two with stroke and Fib, and one with drinking, UA, symptom, TBIL, IBIL, RBC, HGB, and HCT. The correlation coefficient was between −0.422 and 0.413. Methylene bisacrylamide had the highest correlation with sex and was also associated with four of the 49 clinical indicators (sex, D-D, TP, and GLO; Figure 4, Supplementary Table S6).

### 3.5 Predicting metabolic biomarkers and clinical indicators of unstable plaques based on machine learning algorithms

**Figure 5:**
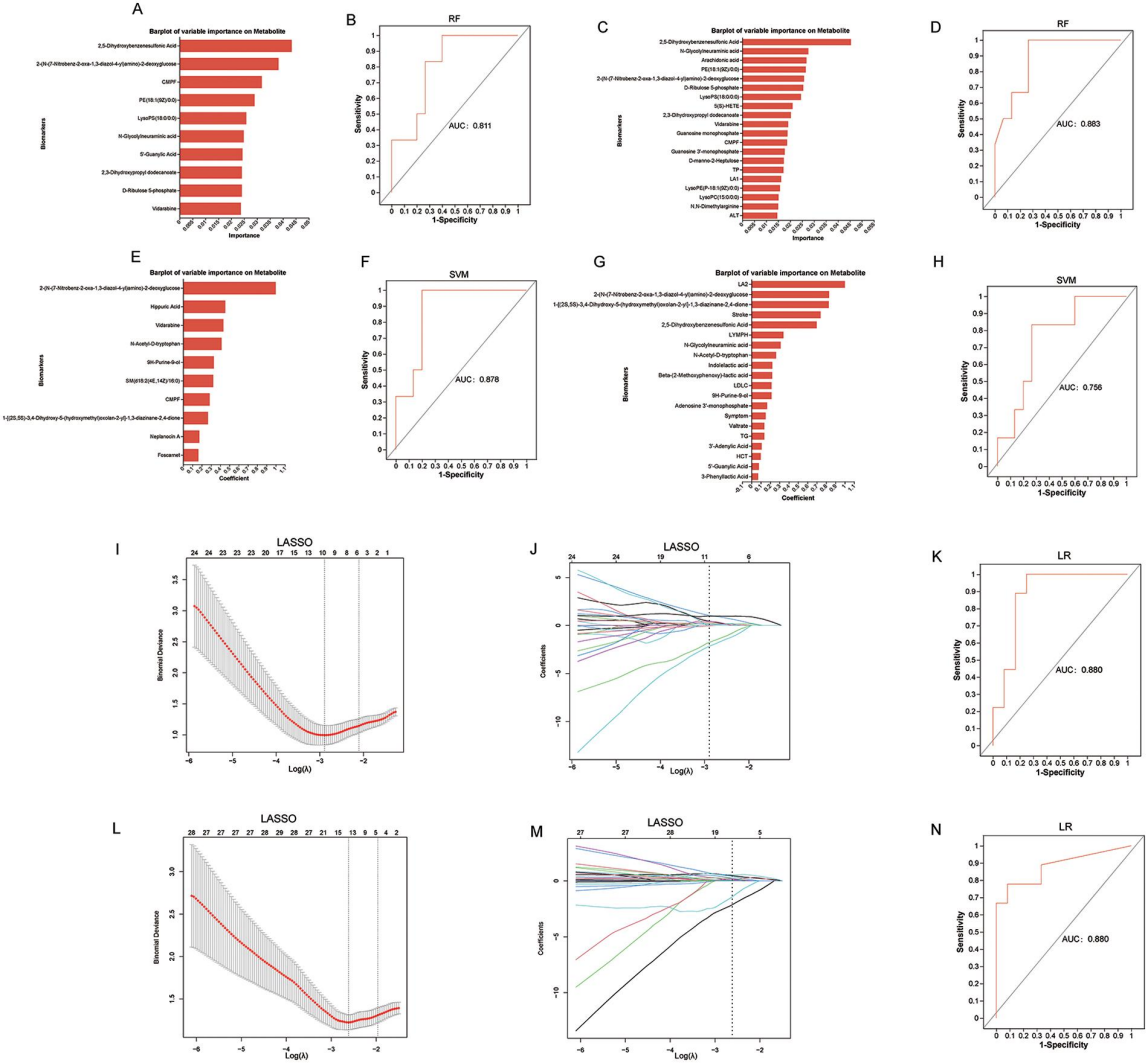
Machine learning algorithms predict metabolic biomarkers and clinical indicators. **(A–D)** Metabolic biomarkers were screened from 98 differential metabolites by random forest (RF) model. **(A)** The 10 metabolites with the highest scores among the 98 differential metabolites in the two groups were predicted based on the RF algorithm. **(B)** ROC curve of 10 metabolites selected by RF model. **(C)** Based on the RF algorithm, predict the top 20 bar charts of 98 differential metabolites and 49 clinical indicators in two groups. **(D)** Area under the ROC curve of the RF model based on these 20 metabolic biomarkers and Clinical indicators. E-H. Metabolic biomarkers were screened from 98 differential metabolites by the Support Vector Machine (SVM) model. **(E)** The 10 metabolites with the highest scores among the 98 differential metabolites in the two groups were predicted based on the SVM algorithm. **(F)** ROC curve of 10 metabolites selected by SVM model. **(G)** Based on the SVM algorithm, predict the top 20 bar charts of 98 differential metabolites and 49 clinical indicators in two groups. **(H)** Area under the ROC curve of the SVM model based on these 20 metabolic biomarkers and Clinical indicators. **(I–K)** Based on the LASSO-Logistic algorithm, metabolic biomarkers were screened from 98 differential metabolites. **(I,J)** 10 metabolic biomarkers associated with unstable plaques predicted by LASSO regression. **(K)** Logistic model ROC curve validation for these 10 screened metabolic biomarkers. **(L–N)** Based on the LASSO + Logistic algorithm, predict metabolic biomarkers and clinical indicators associated with unstable plaques from 98 differential metabolites and 49 clinical indicators. **(L,M)** 12 metabolic biomarkers and 2 clinical indicators associated with unstable plaques predicted by LASSO regression. **(N)** Logistic model ROC curve validation for 12 metabolic biomarkers and 2 clinical indicators. *n*_unstable plaque_ = 27, *n*_stable plaque_ = 40.

To investigate the potential of metabolomics parameters as diagnostic biomarkers, RF, SVM, and LASSO logistic regression classifiers were used to distinguish stable and unstable carotid artery plaques. Different models based on metabolites or combinations of metabolites and clinical indicators were also constructed. The RF model predicted 10 important metabolic biomarkers from 98 differential metabolites (Figure 5A). The model's ROC curve is shown in Figure 5B. After correlating 49 clinical indicators, 17 important metabolic biomarkers and three clinical indicators were predicted (Figure 5C). The model's ROC curve is shown in Figure 5D. The SVM model predicted 10 important metabolic biomarkers from 98 differential metabolites (Figure 5E). The model's ROC plot is shown in Figure 5F. After association with 49 clinical indicators, 13 important metabolic biomarkers and seven clinical indicators were predicted (Figure 5G). The model's ROC curve is shown in Figure 5H. LASSO regression predicted 10 important metabolic biomarkers (Supplementary Table S7) from 98 differential metabolites. Figure 5I shows the cross-validation error curve for the LASSO regression model. The coefficient distribution plot is shown in Figure 5J. A logistic model was constructed based on the above 10 metabolic biomarkers, which were also predicted to be the most important. The model's ROC curve is shown in Figure 5K. After correlating 49 clinical indicators, 12 important metabolic biomarkers and two clinical indicators (Supplementary Table S7) were predicted using LASSO regression. Figure 5L shows the cross-validation error curve for the LASSO regression model. The coefficient distribution plot is shown in Figure 5M. A logistic model was constructed based on the above 12 metabolic biomarkers and two clinical indicators and used to predict seven metabolic biomarkers. The ROC curve for the model is shown in Figure 5N. The diagnostic performance of these machine learning models is shown in Table 1, with an AUC range of 0.756–0.883. A metabolic ensemble of 98 differential metabolites was generated. Then, 30 metabolic biomarkers were screened using RF, SVM, and LASSO logistic regression analysis, identifying 22 metabolic biomarkers associated with unstable plaques after removing duplicate metabolites. In addition, 37 metabolic biomarkers and 12 clinical indicators were screened using RF, SVM, and LASSO logistic regression by associating 49 clinical indicators. Then, 31 metabolic biomarkers and 10 clinical indicators were screened out after removing duplicate values (Figures 5C,G, and Supplementary Table S7). Finally, 14 metabolic biomarkers (Table 2, Supplementary Figure S3) that were most relevant to unstable plaques were identified by intersecting the above 22 metabolic sets with 31 metabolic sets. The distribution of each metabolite's abundance between the two groups is shown in Supplementary Figure 2.

**Table 1:** Diagnostic performance of four machine learning algorithms

Machine learning algorithm models	Classification	Sensitivity	Specificit	ALIC (95% CI)
RF	Metabolites	1.000	0.600	0.811 (0.620–1.000)
	Metabolites + Clinical indicators	1.000	0.730	0.883 (0.737–1.000)
SVM	Metabolites	1.000	0.800	0.878 (0.727–1.000)
	Metabolites + Clinical indicators	0.830	0.730	0.756 (0.532-0.979)
LASSO + LR	Metabolites	1.000	0.750	0.880 (0.722–1.000)
	Metabolites + Clinical indicators	0.780	0.920	0.880 (0.718–1.000)

AUC, area under curve; Cl, confidence interval.

**Table 2:** Metabolic biomarkers based on machine learning prediction

Metabolites	Unstable group		Stable group		Regulate	FC	VIP	*P* value	FDR
	Mean	SD	Mean	SD					
N-Glycolylneuraminic acid	4.681	0.5057	4.058	0.5188	Up	1.1535	3.6292	7.62E-06	0.01131
LysoPS (18:0/0:0)	4.503	0.4716	3.962	0.6013	Up	1.1365	2.9011	0.000208	0.04838
5'-Guanylic Acid	5.813	0.5204	5.083	0.9104	Up	1.1436	3.1636	0.00036	0.04838
1-[(2S,5S)-3,4-Dihydroxy-5-(hydroxymethyl)oxolan-2-yl]-1,3-diazinane-2,4-dione	4.473	0.5299	3.778	0.9784	Up	1.184	4.5081	0.001292	0.1811
N-Acetyl-D-tryptophan	6.4	0.3379	6.642	0.2908	Down	0.9636	2.518	0.002578	0.1811
2-(N-(7-Nitrobenz-2-oxa-1,3-diazol-4-yl)amino)-2-deoxyglucose	4.073	0.5862	3.619	0.5796	Up	1.1254	3.3916	0.002608	0.1811
2,5-Dihydroxybenzenesulfonic Acid	4.246	0.7643	3.66	0.7587	Up	1.1601	3.8007	0.002961	0.1237
SM (d18:2(4E,14Z)/16:0)	6.448	0.1837	6.594	0.1958	Down	0.9779	1.885	0.003111	0.1898
9H-Purine-9-ol	6.596	0.3085	6.814	0.2802	Down	0.968	2.3501	0.003709	0.194
Vidarabine	5.356	0.2697	5.547	0.2445	Down	0.9656	2.2856	0.003785	0.194
2,3-Dihydroxypropyl dodecanoate	4.157	0.07921	4.23	0.1085	Down	0.9827	1.2786	0.004076	0.1999
PE (18:1(9Z)/O:O)	4.702	0.5132	4.331	0.5305	Up	1.0857	3.0679	0.005902	0.2303
CMPF	3.804	0.9657	3.272	0.7254	Up	1.1626	2.9535	0.0124	0.2362
D-Ribulose 5-phosphate	5.473	0.2488	5.035	0.863	Up	1.087	2.0218	0.01278	0.2393

## 4 Discussion

At least 20% of ischemic strokes are thromboembolic and are caused by atherosclerotic plaques at the bifurcation of carotid or internal carotid artery (13, 14). Today, clinical guidelines for the primary and secondary prevention of stroke in patients with carotid artery stenosis caused by atherosclerotic plaques still rely on the patients’ general characteristics (hypertension, smoking status, diabetes, and hypercholesterolemia) and the static measurement of arterial stenosis degree (14). However, the degree of arterial stenosis is not the best estimate of stroke risk, and a new method of assessing stroke risk is needed to improve the risk prediction for individual patients (14). The plaque characteristics are crucial for defining their stability and stroke risk (15–17). Currently, computed tomography (CT), magnetic resonance (MR), coronary computed tomography angiography (CCTA), and positron emission tomography (PET) imaging can identify plaque instability features and directly measure disease activity in coronary arteries and large blood vessels (18). However, the application value of these methods in the early diagnosis of unstable plaques needs to be further validated. It is also important to find new methods to predict unstable plaques for stroke prevention. Metabolomics has emerged as a promising method of identifying potential biomarkers of cerebrovascular disease (19). In the present study, metabolic biomarkers and pathways of unstable plaques were screened using metabolomics with the goal to provide a convenient and low-cost solution for the early diagnosis of unstable plaques.

Metabolomics, together with machine learning algorithms, has become a powerful tool for identifying metabolic biomarkers for disease diagnosis (20). A recent study suggested that metabolomic profiles derived from nuclear magnetic resonance spectroscopy analysis of blood samples can simultaneously inform the risk of multiple diseases (21). Many metabolites in blood samples may have different tissue origins, and the analysis results may be biased. Therefore, unstable and stable carotid artery plaque tissues were selected for metabolomics analysis in the present study. Many studies have reported blood differential metabolic biomarkers and pathways related to risk prediction, early diagnosis, and prognosis of cerebral ischemia (22, 23). Several studies have also elucidated the role of microbial metabolite trimethylamine-N-oxide (TMAO) and lipopolysaccharides in cardiovascular disease and stroke (24, 25), which may trigger atherosclerosis by affecting thrombosis, inflammation, and oxidative stress (26).

In the present study, carotid artery plaques were collected from 67 patients in order to carry out a case-control study to identify 98 differential metabolites. Different models were constructed using machine learning to preliminarily screen 22 important metabolites. To increase the predictive value of plaque metabolomics, metabolomic information was matched with 49 clinical indicators to further predict biomarkers and key clinical indicators associated with unstable plaques. Finally, 14 metabolic biomarkers (with nine upregulated and five downregulated metabolites) and 10 clinical indicators were identified (Supplementary Table S7). These findings to some extent reflect the potential clinical practicality of plaque metabolomics analysis and improve the comprehensive risk assessment of unstable plaques.

N-glycylneuraminic acid (Neu5Gc) is one of the most common forms of sialic acid (27, 28). In mammals, the *de novo* Neu5Gc synthesis pathway begins with the activated precursor cytidine-5′- monophosphate-N acetylneuraminic acid (CMP Neu5Ac), which is catalyzed by CMP Neu5Ac hydroxylase. Very low levels of Neu5Gc can be detected in healthy human tissue (29). Studies have shown that Neu5Gc has a negative impact on the cardiovascular system and leads to the aggravation of atherosclerotic plaques (30). In the present study, Neu5Gc levels were significantly increased in unstable plaque tissues. Since these results were consistent with previous reports, Neu5Gc can serve as a potential metabolic biomarker for unstable plaques.

A variety of lysophospholipids (LPLs) are present in the body, including sphingosine 1-phosphate (S1P), lysophosphatidic acid (LPA), lysophosphatidylserine (LysoPS), and lysophosphatidylinositol (LPI). S1P and LPA have been identified as bioactive substances and are known to be implicated in the pathogenesis of atherosclerosis. In addition, LPA and LysoPS have recently been recognized as bioactive LPLs (31, 32). LysoPS is an emerging lipid mediator, possibly derived from phosphatidylserine, which is exposed to the cell membrane surface during platelet activation (33) or apoptosis (34). It is thought to be implicated in the pathogenesis of acute coronary syndrome (35). Additional studies have shown that LysoPS has several factors involved in the pathogenesis of atherosclerosis. LysoPS enhances the uptake of oxidized low-density lipoprotein, reduces the expression of inflammatory mediators, and alleviates endoplasmic reticulum stress in RAW 264.7 cells. These results suggest that LysoPS has atherogenic properties in foam cell formation and anti-atherosclerotic properties in reducing inflammation, implying that LysoPS has bidirectional properties in the pathogenesis of atherosclerosis (36). In addition, basic studies have shown that LysoPS has a variety of biological activities, such as attenuating the expression of inflammatory mediators in macrophages (36), inhibiting T lymphocyte proliferation (37), and obstructing the development and function of regulatory T lymphocytes (38). However, LysoPS had a significantly increased expression in unstable carotid plaque tissue in the present study. It may also promote the formation of foam cells in unstable plaques, thereby leading to the decline of plaque stability. Therefore, it can be recommended as a potential metabolic marker of unstable plaques.

N-acetyl tryptophan (NAT) is a neurokinin-1 receptor (NK-1R) antagonist that can disrupt the binding of substance P (SP) to NK-1R. In addition, NAT is included in the Neurodegenerative Drug Screening Alliance library consisting of 1,040 compounds compiled by the National Institute of Neurology and Stroke in the United States (39), indicating its potential as a therapeutic agent. There are three NAT isomers: N-acetyl-L tryptophan (L-NAT), N-acetyl-D tryptophan (D-NAT), and N-acetyl-DL tryptophan (DL-NAT) (39). A previous study showed that D-NAT levels in non-small cell lung cancer patients significantly increased after treatment with epidermal growth factor receptor tyrosine kinase inhibitors (EGFR-TKIs) or programmed death 1 (PD-1)/programmed cell death ligand 1 (PD-L1) inhibitors, indicating that D-NAT may have anti-tumor therapeutic effects (40). Metabolic syndrome (MetS) significantly increased the risk of atherosclerotic cardiovascular disease and type 2 diabetes (T2DM), while D-NAT level was significantly reduced in MetS patients. In addition, D-NAT at the physiological level has anti-inflammatory and antioxidant properties, making it an attractive biomarker for the occurrence of MetS due to the critical role of inflammation in MetS (41). The present study results indicated that D-NAT level was significantly reduced in the unstable plaque group compared to the stable plaque group, which strongly suggests its potential as a metabolism-related biomarker.

Iron death is a cell death mechanism characterized by intracellular iron accumulation and lipid peroxidation (42). There are reports that 3-carboxy-4-methyl-5-propyl-2-furanopanoic acid (CMPF) can further induce cell death by increasing reactive oxygen species and lipid peroxidation levels (43). Previous studies have demonstrated that the furan fatty acid metabolite CMPF level increases in patients with gestational diabetes mellitus, T2DM, and impaired glucose tolerance, and damages the pancreatic *β*-cell function. Mechanistically, CMPF directly acts on *β*-cells, leading to impaired mitochondrial function, reduces glucose-induced ATP accumulation, and induces oxidative stress, leading to dysregulation of key transcription factors and ultimately decreasing insulin biosynthesis (44). Liu et al. found that CMPF can promote the development of diabetes by inducing metabolic remodeling to encourage preferential fatty acid use compared to glucose (45). Diabetes is also an important risk factor for atherosclerosis and stroke (46). The present research indicated that CMPF was significantly upregulated in unstable carotid artery plaques, which fully confirms its reliability as a metabolic biomarker.

The present study had some limitations. Only patients who underwent carotid endarterectomy at a single advanced stroke center were enrolled in the study. The sample size was also relatively small, which may have affected statistical analysis and generalization of results. In addition, this was a retrospective study with a potentially lower evidence accuracy compared to that of prospective studies. In addition, the present case-control study design may have been affected by selection bias, making it difficult to determine the chronological order of exposure and disease. Therefore, the data cannot directly determine causal relationships. Nevertheless, the present study was the first to use metabolomics combined with machine learning to predict metabolic biomarkers of unstable carotid artery plaques. It was relatively accurate in identifying metabolic biomarkers for studying the characteristics of unstable plaques and serving as a reference for their early diagnosis.

## 5 Conclusion

In summary, metabolomics analysis of stable and unstable carotid artery plaques was used to identify 14 metabolic biomarkers for early diagnosis their involvement in stroke risk assessment. In addition, it was discovered that metabolites closely related to unstable plaques, especially Neu5Gc, LysoPS, CMPF, and D-NAT, it could help to identify plaque instability and, in turn, contribute to stroke risk stratification. Despite some limitations, the present hypothesis-generating study suggests the existence of metabolic biomarkers and pathways specific of unstable arterial plaques with potential clinical applications upon validation in bigger cohorts.

## Data Availability

The raw data supporting the conclusions of this article will be made available by the authors, without undue reservation.
